# Distance-based assessment of the localization of functional annotations in 3D genome reconstructions

**DOI:** 10.1186/1471-2164-15-992

**Published:** 2014-11-18

**Authors:** Daniel Capurso, Mark R Segal

**Affiliations:** Department of Bioengineering and Therapeutic Sciences, University of California, San Francisco, CA 94107 USA; Department of Epidemiology and Biostatistics, University of California, San Francisco, CA 94107 USA

## Abstract

**Background:**

Recent studies used the contact data or three-dimensional (3D) genome reconstructions from Hi-C (chromosome conformation capture with next-generation sequencing) to assess the co-localization of functional genomic annotations in the nucleus. These analyses dichotomized data point pairs belonging to a functional annotation as “close” or “far” based on some threshold and then tested for enrichment of “close” pairs. We propose an alternative approach that avoids dichotomization of the data and instead directly estimates the significance of distances within the 3D reconstruction.

**Results:**

We applied this approach to 3D genome reconstructions for *Plasmodium falciparum*, the causative agent of malaria, and *Saccharomyces cerevisiae* and compared the results to previous approaches. We found significant 3D co-localization of centromeres, telomeres, virulence genes, and several sets of genes with developmentally regulated expression in *P. falciparum*; and significant 3D co-localization of centromeres and long terminal repeats in *S. cerevisiae*. Additionally, we tested the experimental observation that telomeres form three to seven clusters in *P. falciparum* and *S. cerevisiae*. Applying affinity propagation clustering to telomere coordinates in the 3D reconstructions yielded six telomere clusters for both organisms.

**Conclusions:**

Distance-based assessment replicated key findings, while avoiding dichotomization of the data (which previously yielded threshold-sensitive results).

**Electronic supplementary material:**

The online version of this article (doi:10.1186/1471-2164-15-992) contains supplementary material, which is available to authorized users.

## Background

Recent studies [1–3] employed chromosome conformation capture with next-generation sequencing (Hi-C [4]) to systematically identify genomic regions in physical, three-dimensional (3D) proximity. The resulting contact data lists two genomic positions—each corresponding to a restriction enzyme site—and the frequency with which they were paired-end sequenced together. The smaller the 3D distance between two genomic positions, the larger their interaction frequency should be. Given this relationship, 3D genome reconstructions have been generated from the contact data via constrained optimization for several organisms including *Saccharomyces cerevisiae* [2] and the asexual stages of *Plasmodium falciparum* [3], the causative agent of malaria. Both of these are eukaryotic, haploid, and have relatively small genomes (compared to human). The constraints used in the reconstruction optimization derive from external biological knowledge about genome organization [2, 3].

Both contact data and attendant 3D genome reconstructions are exciting developments because they provide relatively high resolution, genome-wide information on chromosome organization — which previously could only be probed with low-throughput, low-resolution techniques such as fluorescent in situ hybridization (FISH; contrasted in [5]). There is now widespread interest in using this data to gain insight into the 3D nuclear localization of functional genomic annotations (e.g. centromeres, gene ontology (GO) sets). This interest is based on the hypothesis that genome function is linked to its organization [6]. For example, co-regulated genes may be physically co-localized in the nucleus during transcription [7]. Similarly, 3D genome organization likely influences genome stability [8] and the location of DNA breakpoints and gene fusions [8], including those that drive certain cancers [9].

Ay et al [3] recently assessed the co-localization of functional annotations in *P. falciparum* 3D genome reconstructions; however, their approach led to results that were difficult to interpret. Their assessment was performed as follows. For all data point pairs belonging to a given functional annotation, they dichotomized (Euclidean) distances as “close” or “far” based on prescribed thresholds (10%, 20%, or 40% of the nuclear diameter). Then, they assessed enrichment of “close” pairs in that functional annotation using methods developed for contact data [6]. In the results of this analysis, some functional annotations were significant across all thresholds; however, many functional annotations were significant for only one (or two) threshold(s) but not the other(s). Further, there was often no consistent relationship with respect to threshold. This makes interpretation difficult, especially since it is not obvious what constitutes a good choice for a biologically meaningful threshold. We refer to this approach as “dichotomized distance enrichment” throughout the paper.

Similar analyses have been performed in *S. cerevisiae* [6, 10, 11] using contact data rather than the 3D genome reconstruction. Here, pairs of data points belonging to a functional annotation were dichotomized as “close” if they were observed together (i.e. if their interaction frequency passed (False Discovery Rate [12]) filtering); otherwise they were “far”. Then, the enrichment of “close” pairs in the functional annotation was tested. We refer to this approach as “dichotomized contact enrichment” throughout the paper.

Rather than dichotomizing the data, we propose directly assessing the significance of distances derived from the 3D reconstruction. This approach is potentially an improvement over previous analyses since it avoids dichotomization of distances (which could incur information loss) and does not require (arbitrary) thresholding or tuning. For a given functional annotation, we computed the median of pairwise Euclidean distances (MPED) between data points belonging to that functional annotation and then assessed the significance of this test statistic by resampling. We also expanded to two-tailed analyses to enable tests for *dispersion* of functional annotations since, for example, localization near the nuclear periphery is functionally relevant [13]. Our approach provided novel findings, replicated key results from prior analyses and provided unambiguous inference for functional annotations that previously reported significance levels that varied by dichotomization threshold. We refer to our approach as “MPED assessment” throughout the paper.

## Results

**Figure 1:**
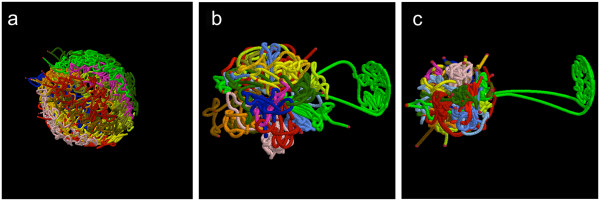
**3D genome reconstructions. (a)** *P. falciparum* Ring stage 3D genome reconstruction. *S. cerevisiae* 3D genome reconstructions from **(b)** HindIII or **(c)** EcoRI restriction enzyme libraries.

We performed MPED assessment of functional annotation localization in 3D genome reconstructions (see *Methods*) for *P. falciparum* Ring stage [3] and *S. cerevisiae* [2] from two different restriction enzyme libraries, HindIII and EcoRI (Figure 1). We also tested dichotomized contact enrichment (as in [6]; see *Methods*) and compared the results. Results for dichotomized distance enrichment have been reported in detail previously (see “Supplemental Information” from [3]).

### 3D localization of *P. falciparum*functional genomic annotations

For *P. falciparum* Ring stage, we assessed the localization of the following functional annotations: centromeres, telomeres, virulence (VRSM) genes, rDNAs, and 15 clusters of genes with developmentally regulated expression [3, 14]. We used normalized [15] *P. falciparum* Ring stage contact data and the (extensively validated) 3D genome reconstruction inferred from these data [3].

Centromeres, telomeres, and VRSM genes were significantly co-localized under MPED assessment (Table 1). These functional annotations were also significantly co-localized under dichotomized contact enrichment (Table 1) and under dichotomized distance enrichment at all three thresholds examined (10%, 20%, or 40% of the nuclear diameter; see “Supplemental Information” from [3]). Furthermore, experimental FISH data supports the nuclear clustering of telomeres in *P. falciparum* [16, 17].

**Table 1:** **Assessment of the 3D localization of functional annotations in** ***P. falciparum*** **Ring stage**

Functional annotation	MPED	Contact enrichment
	q-values	q-values
Centromeres	**6.0e-05**	**8.4e-05**
Telomeres	**6.0e-05**	**8.4e-05**
VRSM (all)	**6.0e-05**	**8.4e-05**
VRSM (subtelomeric)	**6.0e-05**	**8.4e-05**
VRSM (internal)	**1.6e-04**	**8.4e-05**
rDNA genes	0.42	0.10
Cluster 1	0.73 ↓	0.17
Cluster 2	**4.4e-02**	0.70 ↓
Cluster 3	0.18	0.45 ↓
Cluster 4 (Ring)	**6.0e-05**	**1.0e-02**
Cluster 5 (Ring)	0.24	0.45 ↓
Cluster 6 (Ring	**6.0e-05**	0.70 ↓
Cluster 7 (Ring)	**6.0e-05**	0.39 ↓
Cluster 8	**4.0e-02**	0.86 ↓
Cluster 9	**1.0e-02**	0.39 ↓
Cluster 10	**2.1e-03**	0.81
Cluster 11	0.10	0.74 ↓
Cluster 12	**9.2e-03**	0.11 ↓
Cluster 13	6.5e-02	0.44 ↓
Cluster 14	0.11	0.70 ↓
Cluster 15	5.2e-02	0.81 ↓

*MPED*: the median of pairwise Euclidean distances in the 3D reconstruction. *Contact enrichment*: enrichment of dichotomized “close” pairs in the Hi-C contact data. Bold indicates q-value <0.05. Down arrow indicates dispersion (otherwise co-localization). All functional annotations that were tested are included. “Cluster N” refers to genes with life cycle -regulated expression, which were clustered in (Le Roch et al [14]). Clusters that have high gene expression in the Ring stage are indicated in parentheses.

Eight out of 15 clusters of genes with developmentally regulated expression (including several with Ring stage expression) were significantly co-localized under MPED assessment, but only 1 was significantly co-localized under dichotomized contact enrichment (Table 1). Of the 8 expression clusters significantly co-localized under MPED assessment, only 2 were significant across all three thresholds under dichotomized distance enrichment (see “Supplemental Information” from [3]); the other 6 had threshold-dependent significance under dichotomized distance enrichment. In the *Discussion*, we comment on why assessing localization at the 3D reconstruction level (with MPED) may reveal significant co-localization for some functional groups that was not detected using contact level data.

### 3D localization of *S. cerevisiae*functional genomic annotations

For *S. cerevisiae*, we assessed the localization of 264 GO terms and 17 other functional annotations, including centromeres, telomeres, retrotransposon long terminal repeats (LTRs), classes of non-coding RNAs, classes of replication origins, classes of DNA breakpoints, and classes of cell cycle -regulated genes (full list in *Methods*). We report functional annotations that were significant under MPED assessment with both restriction enzyme libraries (HindIII and EcoRI) or significant with both libraries under dichotomized contact enrichment.

There is no indication that the *S. cerevisiae* Hi-C data was normalized in previous studies [2, 6] prior to generating the 3D genome reconstructions or assessing functional annotation localization: the original study [2] preceded the formalization of Hi-C data normalization pipelines [15, 18, 19] that redress biases due to factors such as fragment length, GC content and mappability. Accordingly, we normalized the *S. cerevisiae* Hi-C contact data (see *Methods*) and then generated new reconstructions, as in [2], from the normalized contact data (Figure 1) before assessing functional annotation localization.

Centromeres and LTRs were significantly co-localized under MPED assessment and under dichotomized contact enrichment (Table 2). Previous analyses of this *S. cerevisiae* Hi-C data also found significant co-localization of centromeres [6] and LTRs [20]. Furthermore, experimental FISH data support the nuclear clustering of centromeres [21] and LTRs [22] in *S. cerevisiae*. Several GO terms that map to LTRs (e.g., retrotransposon nucleocapsid, transposition) were also significantly co-localized under both analyses but are not included in Table 2 because of the redundancy in the mapping.

**Table 2:** **Assessment of the 3D localization of functional annotations in** ***S. cerevisiae***

Functional annotation	MPED q-values		Contact enrichment q-values	
	HindIII	EcoRI	HindIII	EcoRI
Centromeres	**4.0e-04**	**3.7e-04**	**2.8e-03**	**5.6e-03**
Long terminal repeats	**4.0e-04**	**3.7e-04**	**2.8e-03**	**1.9e-02**
Telomeres	0.86 ↓	0.13 ↓	**5.0e-02**	**5.6e-03**

*MPED*: the median of pairwise Euclidean distances in the 3D reconstruction. *Contact enrichment*: enrichment of dichotomized “close” pairs in the Hi-C contact data. Bold indicates q-value <0.05. Down arrow indicates dispersion (otherwise co-localization). Functional annotations are included if they were significant for both restriction enzyme libraries (HindIII and EcoRI) in either analysis.

Telomeres were significantly co-localized under dichotomized contact enrichment, but not under MPED assessment (Table 2). Experimental FISH data support nuclear clustering of *S. cerevisiae* telomeres [23, 24]. In the *Discussion*, we comment on why assessing localization at the 3D reconstruction level (with MPED) may not detect significant co-localization for some functional groups that were detected at the contact data level (particularly the difficulty of generating a null distribution for telomeres).

The previous study that analyzed *S. cerevisiae* functional annotation localization under dichotomized contact enrichment reported significant co-localization of certain functional groups (e.g., early replication origins (Clb5 and Rad53), and tRNAs) [6] that were not replicated in our analysis under dichotomized contact enrichment. This difference may be due to our testing a much larger number of functional groups (and the corresponding multiplicity correction) and/or our normalization of the data prior to assessment. Experimental FISH data supports tRNA clustering in *S. cerevisiae* [25]. Under dichotomized contact enrichment, our q-values for tRNAs were 2.4e-02 (HindIII) and 0.55 (EcoRI). Under MPED assessment, our q-values for tRNAs were 0.64 (HindIII) and 2.0e-03 (EcoRI).

### Generating a null referent distribution

In our MPED assessment of functional annotation localization above, we generated a null referent distribution by resampling points from the same chromosome as observed (i.e. preserving the chromosome structure of the data).

An alternative approach is to resample preserving the distance that a data point is from the center of the nucleus (within a range), but not preserving the chromosome structure. Such a resampling scheme may detect functional groups that are co-localized given the Rabl configuration of the *S. cerevisiae* 3D genome reconstructions [2]. To perform such a resampling scheme, we divided the radius of the nucleus into fifths and created a series of concentric spheres at each partition. Points were then resampled from the 3D annulus (ring) between concentric spheres. The results under MPED assessment with annulus resampling were similar to those with chromosome resampling for both organisms (Additional file 1).

### Affinity propagation clustering applied to 3D telomere coordinates

**Figure 2:**
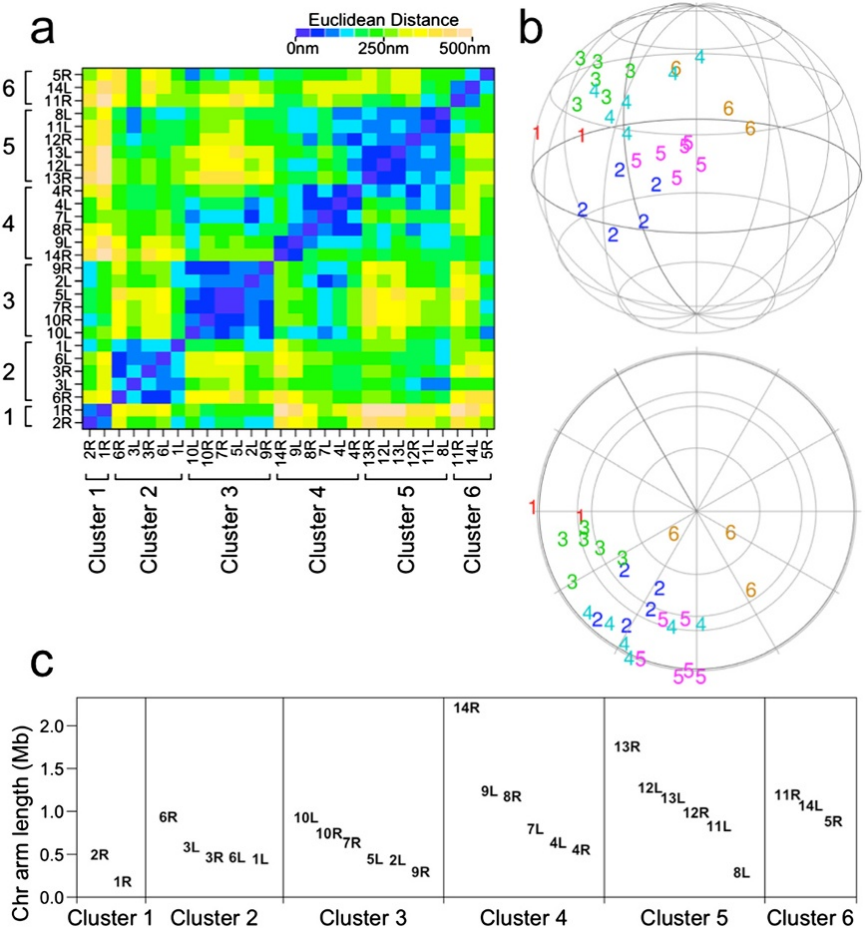
**Affinity Propagation clustering applied to 3D telomere coordinates for** ***P. falciparum*** **Ring stage. (a)** Heat map of Euclidean distances between telomeres. The clustering is indicated. **(b)** Positions of telomeres in the 3D reconstruction plotted as the cluster number. *Upper*: side view. *Lower*: top view, a 90-degree rotation forward about the z-axis relative to the side view. **(c)** The chromosome arm lengths of telomeres in each cluster.

**Figure 3:**
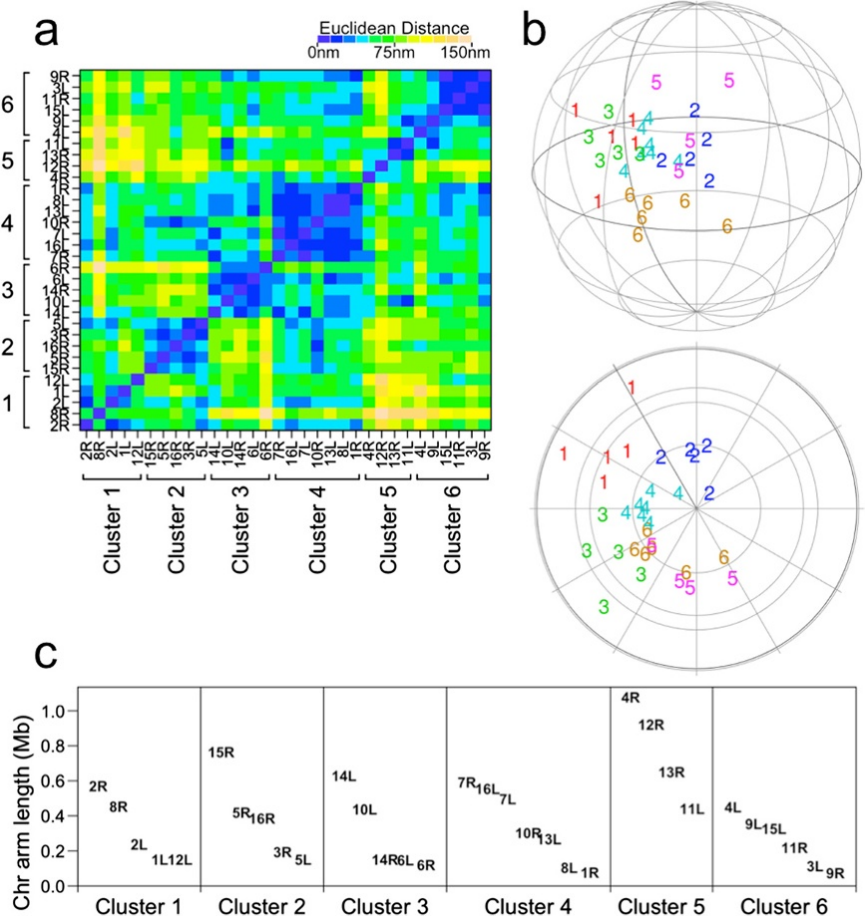
**Affinity Propagation clustering applied to 3D telomere coordinates for** ***S. cerevisiae*** (**HindIII**). **(a)** Heat map of Euclidean distances between telomeres. The clustering is indicated. **(b)** Positions of telomeres in the 3D reconstruction plotted as the cluster number. *Upper*: side view. *Lower*: top view, a 90-degree rotation forward about the z-axis relative to the side view. **(c)** The chromosome arm lengths of telomeres in each cluster.

Experimental FISH data indicate that telomeres form 4 to 7 clusters in *P. falciparum* [16, 17] and 3 to 7 clusters in *S. cerevisiae* [23, 24]. To determine if we could recapitulate this property of telomere organization from the 3D genome reconstructions (and to identify which telomeres are close to each other) we applied affinity propagation (AP) clustering [26] to telomere coordinates in the 3D genome reconstructions. Unlike many other clustering algorithms (e.g. *k*-means) where the number of clusters needs to be specified from the outset, AP clustering optimizes the number of clusters within the algorithm. Applying AP clustering yielded 6 telomere clusters for both *P. falciparum* (Figure 2) and *S. cerevisiae* (Figure 3), consistent with the FISH data. This also revealed which telomeres are close to each other in the 3D genome reconstructions (Figures 2 and 3).

## Discussion

In this study, we propose using MPED to assess functional annotation localization and applied this approach to *P. falciparum* and *S. cerevisiae* 3D genome reconstructions. We argue that, if functional annotation localization assessment is to be performed at the 3D genome reconstruction level, then MPED assessment offers advantages over dichotomized distance enrichment [3] because it avoids dichotomization of the data (which could incur information loss) and does not require (arbitrary) thresholding or tuning thereby providing unambiguous results.

However, as with any statistic and associated inferential assessment, MPED embodies specific choices and assumptions. For the statistic, we have employed the *median* (because of its robustness and resistance properties) of *all* pairwise distances (because this does not require tuning as, for example, would be necessary with *k* nearest neighbor distances). Evaluation of alternative formulations (mean rather than median; *k* nearest neighbor distances rather than pairwise distances) had comparable results (when *k* ≥2). For inferential assessment, we have used two approaches to generating null referent distributions (as described above); other potentially organism-specific possibilities could be entertained. A strength of methods imposing dichotomization is that obtaining reasonable referent distributions is relatively straightforward.

There are other putative advantages of assessing functional annotation localization at the 3D reconstruction level: (i) while the contact data is inherently pairwise, the 3D reconstructions exploit higher order relationships; (ii) the 3D location of sites for which there is missing contact data is readily determined from neighbouring points in the reconstruction because of chromatin contiguity; and (iii) biological and biophysical constraints about genome organization are imposed (e.g. avoidance of steric clashes). Thus, emergent properties of the 3D reconstructions may reveal significant co-localization of some functional annotations that were not co-localized in the (pairwise) contact data (e.g. *P. falciparum* gene expression clusters).

The advantage of assessing functional annotation localization at the contact data level is that resampling to generate a null distribution makes recourse only to chromosome labels, while at the 3D reconstruction level, resampling makes recourse to the (more complex) chromatin structure. The 3D reconstructions for *S. cerevisiae* have low chromatin density near the nuclear periphery and large chromatin voids in the nucleus (Figure 1). Given that *S. cerevisiae* telomeres are in the periphery, resampling making recourse to the chromatin structure thus samples points from more internally than the telomeres de facto (even with annulus resampling), which may make it difficult to detect co-localization. Resampling points *without* making recourse to the chromatin structure (i.e. any X,Y,Z coordinate within an annulus) would not be stringent enough. *S. cerevisiae* telomeres may be co-localized given a spherical 3D nucleus (and ignoring the chromatin structure within); however, MPED assessment does not detect significant co-localization of *S. cerevisiae* telomeres by generating a null distribution by resampling points making recourse to the (complex) chromatin structure.

It is important to note that there are caveats to the use of Hi-C data (whether at the contact data level or 3D genome reconstruction level). Most current Hi-C data represents averages over many cells. The first example of single cell Hi-C in mouse has recently been reported [27]; however, a 3D mammalian genome reconstruction has not yet been generated for computational reasons. Mammalian Hi-C analysis is complicated further by diploid genomes, though methods related to Hi-C have been developed for deconvolving sequence data for homologous chromosomes [28]. Finally, Hi-C is a snapshot of highly dynamic chromatin organization; these dynamics are important to understand, but difficult to capture. For the 3D reconstruction-based approach to be meaningful requires that the reconstruction provides an adequate representation of dynamics and between-cell variation. Methods for making such assessments and devising and contrasting reconstruction algorithms are active research areas [29–31].

In the current study, we assessed the 3D localization of genomic annotations (point data). Each data point has an X,Y,Z coordinate; co-localization is assessed by estimating the significance of distances between points. In future research, we will expand to assessing the 3D localization of continuous, functional genomic data – for example, by overlying chromatin immunoprecipitation sequencing (ChIP-seq) peak height on top of the 3D reconstructions. While our current research provides a framework for such an analysis, future research will require developing and/or applying methodology suited to detect co-localization of data that has an X,Y,Z coordinate paired with a continuous outcome (peak height).

## Conclusions

When assessing functional annotation localization at the 3D reconstruction level: MPED assessment, as proposed and applied here, offers advantages over the existing approach (dichotomized distance enrichment). MPED assessment replicated key findings from previous analyses, as well as providing novel results, and provided unambiguous significance estimates for functional annotations that previously had significance levels that varied by threshold.

## Methods

### *P. falciparum*data and annotations

The *P. falciparum* Ring stage contact data and 3D reconstruction were obtained at: <http://noble.gs.washington.edu/proj/plasmo3d/>. This data has already been normalized and filtered [3]. Various functional annotations were assessed: centromeres, telomeres, rDNA genes, VRSM genes, and developmentally regulated gene expression clusters [14]. All of these annotations are available at the same link as for the *P. falciparum* contact data (above).

### *S. cerevisiae*data and annotations

*S. cerevisiae* contact data (pre-FDR, no masking) for HindIII and EcoRI [2] were obtained at: < http://noble.gs.washington.edu/proj/yeast-architecture/sup.html>. We normalized this contact data for GC content, mappability, and fragment length by applying HiCNorm [19] genome-wide (chromosome by chromosome). We then filtered to retain the top contacts by interaction frequency. We generated new 3D genome reconstructions [2] for HindIII and EcoRI based on this normalized and filtered contact data.

Various functional annotations were assessed. Annotations for centromeres, telomeres, retrotransposon long terminal repeats (LTRs), transfer RNAs (tRNAs) and small nucleolar RNAs (snoRNAs) were obtained from the Table Browser of the UCSC Genome Browser [32]. Annotations for early Clb5-independent replication origins, late Clb5-dependent replication origins, early Rad53-regulated origins, and late Rad53-regulated origins from [33] were obtained at: < http://noble.gs.washington.edu/proj/yeast-architecture/sup.html>. Gene Ontology (GO) term annotations were obtained from the Gene Ontology Website [34] and corresponding gene coordinates were obtained from the Table Browser of the UCSC Genome Browser [32]. We filtered GO terms by membership: 264 terms with between 25 and 120 genes were retained for analysis. Cell cycle-regulated genes (5 clusters of genes with expression that peaks during M/G1, G1, S, S/G2, or G2/M) from [35] were obtained at: < http://genome-www.stanford.edu/cellcycle/data/rawdata/>. Annotations for DNA breakpoints from [33] were obtained at: < http://gbe.oxfordjournals.org/content/1/350/suppl/DC1>. Genomic positions in these files were for the sc1 assembly of the *S. cerevisiae* genome, so we converted to sc2 assembly positions using the Batch Coordinate Conversion (liftover) tool from the UCSC Genome Browser [36]. Three categories of DNA breakpoints were used in the analyses: experimentally-induced (mutagenized) breakpoints, evolutionary breakpoints compared to *Kluyveromyces waltii*, and evolutionary breakpoints compared to the hypothetical/inferred ancestor that *S. cerevisiae* and *K. waltii* share [33, 37].

### MPED assessment

The 3D genome reconstruction data consists of a series of “beads” spaced throughout the linear genome. Each bead has a genomic position and a 3D coordinate (X,Y,Z). To map functional annotations to the 3D reconstruction data, we assigned each centromere, for example, to its nearest bead in linear, genomic space.

We assessed functional annotation localization at the 3D genome reconstruction level as follows. We employed the median of pairwise Euclidean distances (MPED) –applied interchromosomally, in order to avoid detection of annotations simply clustered in linear, genomic space [6]. To estimate MPED significance, we generated a null referent distribution by resampling 1e05 times with preservation of the chromosome structure of the data. For example, for centromeres—where there is one centromere per chromosome—we randomly selected one bead from each chromosome during each resampling, and computed and saved the MPED.

Results from preservation of the chromosome *arm* structure of the data (not shown) were very similar to those obtained from preserving the chromosome structure of the data. We also tried preserving the annulus structure of the data – in other words, preserving the approximate distance that a bead is from the center of the nucleus, but not preserving the chromosome structure of the data. For annulus resampling, we divided the radius into fifths and created concentric spheres at each partition; we then resampled beads from the appropriate annulus (ring) between concentric spheres.

We estimated p-values as follows. When the test statistic was greater than the mean of the null referent distribution (of MPEDs from resampling), the p-value was based on comparison to the upper tail of the distribution (and, if significant, would indicate dispersion). When the statistic was less than the mean of the null referent distribution, the p-value was based on comparison to the lower tail of the distribution (and, if significant, would indicate co-localization). We used False Discovery Rate (FDR) [12] for multiple testing corrections and accepted an FDR q-value of <0.05 as significant.

### Dichotomized contact enrichment

The contact data lists two genomic positions— each corresponding to restriction enzyme site (or bin, if the data is binned) — and the frequency with which the two interact (are sequenced together). The normalized contact data was filtered to retain only the top contacts by interaction frequency [2]. We mapped functional annotations to the filtered contact data as in [2]: for a given centromere, for example, all restriction sites within a window are assigned to that centromere (along with the attendant contact data). The window sizes were 5 kb for *S. cerevisiae* and 10 kb for *P. falciparum*, in line with the resolution/binning of the respective 3D reconstructions [2, 3].

To assess functional annotation localization from the contact data, we used dichotomized contact enrichment [6]. Pairs of elements belonging to a functional annotation were considered “close” if the restriction enzyme sites to which they map were present together in the filtered contact data. The test statistic is the (genome-wide) ratio of the number of observed, interchromosomal “close” pairs (*k*) to the number of possible, interchromosomal pairs (*m*). To estimate *k*:*m* significance, we generated a null referent distribution by resampling 1e05 times as follows. For each chromosome, we resampled the same number of restrictions sites as were assigned on that chromosome and then computed and saved the statistic. We estimated p-values by comparing the test statistic to the null referent distribution, as described above for the reconstruction-based assessment. Our analysis differs from [6] in that we perform a two-tailed assessment. We again used FDR for multiple testing correction with a q-value of <0.05 accepted as significant.

### Ethics

This research utilized publicly available datasets. This research did not utilize data for human subjects or vertebrates.

## Electronic supplementary material

Additional file 1: Table S1: Comparison of resampling schemes for distance-based assessment of the localization of functional annotations in *P. falciparum* Ring stage. **Table S2.** Comparison of resampling schemes for distance-based assessment of the localization of functional annotations in *S. cerevisiae*. (DOCX 63 KB)
