# Supplementary material for: Distance-based assessment of the localization of functional annotations in 3D genome reconstructions
Source: BMC Genomics. 2014 Nov 18;15(1):992. doi: 10.1186/1471-2164-15-992 (PMC4254257; doi:10.1186/1471-2164-15-992)
Supplement: Supplementary file 1 — Additional file 1: Table S1: Comparison of resampling schemes for distance-based assessment of the localization of functional annotations in P. falciparum Ring stage. Table S2. Comparison of resampling schemes for distance-based assessment of the localization of functional annotations in S. cerevisiae. (DOCX 63 KB) [file 12864_2014_6703_MOESM1_ESM.docx]

**Supplementary data for manuscript:**

**Distance-based assessment of the localization of functional annotations in 3D genome reconstructions**

Daniel Capurso^1^, Mark R Segal^2*^

^1^Department of Bioengineering and Therapeutic Sciences,

^2^Department of Epidemiology and Biostatistics,

University of California, San Francisco, California, United States of America

^*^Corresponding author

Email addresses:

DC: [daniel.capurso@ucsf.edu](mailto:daniel.capurso@ucsf.edu)

MRS: [mark.segal@ucsf.edu](mailto:mark@biostat.ucsf.edu)

## Table S1. Comparison of resampling schemes for distance-based assessment of the localization of functional annotations in *P. falciparum* Ring stage.

Points were resampled within the same chromosome or within the same annulus. Gray shading indicates q-value <0.05. Down arrow indicates dispersion (otherwise co-localization). All functional annotations that were tested are included. “Cluster N” refers to genes with life cycle -regulated expression, which were clustered in (Le Roche et al (2003)). Clusters that have high gene expression in the Ring stage are indicated in parentheses.

| **Functional annotation** | **Chromosome resampling**  **q-values** | **Annulus (Rabl)**  **resampling**  **q-values** |
| --- | --- | --- |
| Centromeres | 6.0e-05 | 7.0e-05 |
| Telomeres | 6.0e-05 | 7.0e-05 |
| VRSM (all) | 6.0e-05 | 7.0e-05 |
| VRSM (subtelomeric) | 6.0e-05 | 7.0e-05 |
| VRSM (internal) | 1.6e-04 | 7.0e-05 |
| rDNA genes | 0.42 | 0.22 |
| Cluster 1 | 0.73 ↓ | 0.77 ↓ |
| Cluster 2 | 4.4e-02 | 2.6e-03 |
| Cluster 3 | 0.18 | 3.3e-04 |
| Cluster 4 (Ring) | 6.0e-05 | 2.6e-04 |
| Cluster 5 (Ring) | 0.24 | 3.5e-03 |
| Cluster 6 (Ring | 6.0e-05 | 7.0e-05 |
| Cluster 7 (Ring) | 6.0e-05 | 7.9e-02 |
| Cluster 8 | 4.0e-02 | 0.45 |
| Cluster 9 | 1.0e-02 | 2.7e-03 |
| Cluster 10 | 2.1e-03 | 6.3e-03 |
| Cluster 11 | 0.10 | 2.5e-02 |
| Cluster 12 | 9.2e-03 | 1.2e-04 |
| Cluster 13 | 6.5e-02 | 1.0e-03 |
| Cluster 14 | 0.11 | 3.8e-04 |
| Cluster 15 | 5.2e-02 | 0.22 |

## Table S2. Comparison of resampling schemes for distance-based assessment of the localization of functional annotations in *S. cerevisiae*.

Points were resampled within the same chromosome or within the same annulus. Gray shading indicates q-value <0.05. Down arrow indicates dispersion (otherwise co-localization). Functional annotations from Table 2 are shown.

| **Functional annotation** | **Chromosome**  **resampling q-values** | | **Annulus (Rabl)**  **resampling q-values** | |
| --- | --- | --- | --- | --- |
|  | **HindIII** | **EcoRI** | **HindIII** | **EcoRI** |
| Centromeres | 4.0e-04 | 3.7e-04 | 5.1e-04 | 0.21 |
| Long terminal repeats | 4.0e-04 | 3.7e-04 | 5.1e-04 | 4.0e-04 |
| Telomeres | 0.86 ↓ | 0.13 ↓ | 0.88 ↓ | 0.39 ↓ |
